# The effect of low- versus normal-pressure pneumoperitoneum during laparoscopic colorectal surgery on the early quality of recovery with perioperative care according to the enhanced recovery principles (RECOVER): study protocol for a randomized controlled study

**DOI:** 10.1186/s13063-020-04496-8

**Published:** 2020-06-17

**Authors:** Kim I. Albers, Fatih Polat, Ivo F. Panhuizen, Marc M. J. Snoeck, Gert-Jan Scheffer, Hans D. de Boer, Michiel C. Warlé

**Affiliations:** 1 Department of Surgery and Anesthesiology, Radboud University Medical Center, Geert Grooteplein Zuid 10, 6525 GA Nijmegen, The Netherlands; 2 Department of Surgery, Canisius Wilhelmina Hospital, Weg door Jonkerbos 100, 6532 SZ Nijmegen, The Netherlands; 3 Department of Anesthesiology, Canisius Wilhelmina Hospital, Weg door Jonkerbos 100, 6532 SZ Nijmegen, The Netherlands; 4 Department of Anesthesiology, Radboud University Medical Center, Geert Grooteplein Zuid 10, 6525 GA Nijmegen, The Netherlands; 5 Department of Anesthesiology, Martini General Hospital, Van Swietenplein 1, 9728 NT Groningen, The Netherlands; 6 Department of Surgery, Radboud University Medical Center, Geert Grooteplein Zuid 10, 6525 GA Nijmegen, The Netherlands

**Keywords:** Pneumoperitoneum, Intra-abdominal pressure, Deep neuromuscular block, Laparoscopy, Colorectal, Laparoscopic surgery, Quality of recovery, Rocuronium, Sugammadex

## Abstract

**Background:**

There is increasing evidence for the use of lower insufflation pressures during laparoscopic surgery. Deep neuromuscular blockade allows for a safe reduction in intra-abdominal pressure without compromising the quality of the surgical field. While there is considerable evidence to support superior surgical conditions during deep neuromuscular blockade, there is only a limited amount of studies investigating patient outcomes. Moreover, results are not always consistent between studies and vary between different types of laparoscopic surgery. This study will investigate the effect of low-pressure pneumoperitoneum facilitated by deep NMB on quality of recovery after laparoscopic colorectal surgery.

**Methods:**

The RECOVER study is a multicenter double-blinded randomized controlled trial consisting of 204 patients who will be randomized in a 1:1 fashion to group A, low-pressure pneumoperitoneum (8 mmHg) facilitated by deep neuromuscular blockade (post tetanic count of 1–2), or group B, normal-pressure pneumoperitoneum (12 mmHg) with moderate neuromuscular blockade (train-of-four response of 1–2). The primary outcome is quality of recovery on postoperative day 1, quantified by the Quality of Recovery-40 questionnaire.

**Discussion:**

Few studies have investigated the effect of lower insufflation pressures facilitated by deep neuromuscular blockade on patient outcomes after laparoscopic colorectal procedures. This study will identify whether low pressure pneumoperitoneum and deep neuromuscular blockade will enhance recovery after colorectal laparoscopic surgery and, moreover, if this could be a valuable addition to the Enhanced Recovery After Surgery guidelines.

**Trial registration:**

EudraCT 2018-001485-42. Registered on April 9, 2018. Clinicaltrials.govNCT03608436. Registered on July 30, 2018.

## Background

There is increasing evidence for the use of lower insufflation pressures during laparoscopic surgery [1–5]. Consensus guidelines recommend using the lowest intra-abdominal pressure with an adequate view of the surgical field [6, 7]. This level varies between patients based on many factors that influence compliance of the abdominal wall. Deep neuromuscular blockade (NMB) allows for a safe reduction in intra-abdominal pressure without compromising the quality of the surgical field. Our research group has performed multiple studies investigating surgical conditions in living kidney donors. In a randomized controlled trial in laparoscopic donor nephrectomy patients, we show that compared to moderate NMB, deep NMB allows for lower mean insufflation pressures while maintaining significantly better surgical conditions on the Leiden-Surgical Rating Scale (L-SRS, displayed in Table 1) [9]. Similar results have been reported for surgical conditions during other abdominal laparoscopic procedures. Meta-analysis shows that compared to moderate NMB, deep NMB improves laparoscopic surgical space conditions with a mean difference of 0.65 (95% CI 0.47–0.83) on the L-SRS scale [10]. While there is considerable evidence to support superior surgical conditions during deep NMB, only a limited amount of studies investigate patient outcomes. Moreover, results are not always consistent between studies. Understandably, results can vary between different types of laparoscopic surgery. As carefully outlined by Fuchs-Buder and colleagues in their review, the location of the surgical field (e.g., is the space confined, encompassed by muscular tissue or close to the diaphragm) will have a great influence on the effect of lower pneumoperitoneum pressures or deep neuromuscular block on surgical conditions [11]. A systematic review by Madsen et al. provides evidence for the use of deep NMB during laparoscopic cholecystectomy, prostatectomy, and nephrectomy [12]. Results of Torensma et al. support deep NMB during laparoscopic bariatric surgery [13]. Only a few studies have investigated the influence of lower intra-abdominal pressure or deep NMB on surgical conditions and patient outcomes for colorectal laparoscopic surgery. Koo et al. found less abrupt increases in intra-abdominal pressure with deep NMB as compared to moderate NMB [14]. Cho and colleagues found decreasing intra-abdominal pressure during laparoscopic colorectal surgery provides no cardiopulmonary benefits, regardless of the level of NMB [15]. Diaz-Cambronero et al. used an individualized strategy to titrate intra-abdominal pressure and found 78% of colorectal surgeries could be completed at low pressure (8 mmhg) [16]. Kim et al. found that compared to moderate NMB, deep NMB allows titration to lower insufflation pressures (9.3 mmHg versus 12 mmHg) while maintaining the surgical field. Additionally, they report lower postoperative pain scores, a lower incidence of postoperative shoulder tip pain and faster recovery of bowel function in the deep NMB group [17]. Whether low pressure pneumoperitoneum and deep NMB improve early quality of recovery after laparoscopic colorectal surgery is still unknown. We hypothesize low pressure pneumoperitoneum facilitated by deep NMB will enhance postoperative quality of recovery.

**Table 1:** Leiden-Surgical Rating Scale [8]

	Scale	Description
1	Extremely poor conditions	The surgeon is unable to work due to coughing or due to the inability to obtain a visible laparoscopic field because of inadequate muscle relaxation. Additional muscle relaxants must be given.
2	Poor conditions	There is a visible laparoscopic field but the surgeon is severely hampered by inadequate muscle relaxation with continuous muscle contractions and/or movements with the hazard of tissue damage. Additional muscle relaxants must be given.
3	Acceptable conditions	There is a wide visible laparoscopic field but muscle contractions and/or movements occur regularly causing some interference with the surgeon’s work. There is the need for additional muscle relaxants to prevent deterioration.
4	Good conditions	There is a wide laparoscopic working field with sporadic muscle contractions and/or movements. There is no immediate need for additional muscle relaxants unless there is the fear for deterioration.
5	Optimal conditions	There is a wide visible laparoscopic working field without any movement or contractions. There is no need for additional muscle relaxants.

## Methods

**Fig. 1:**
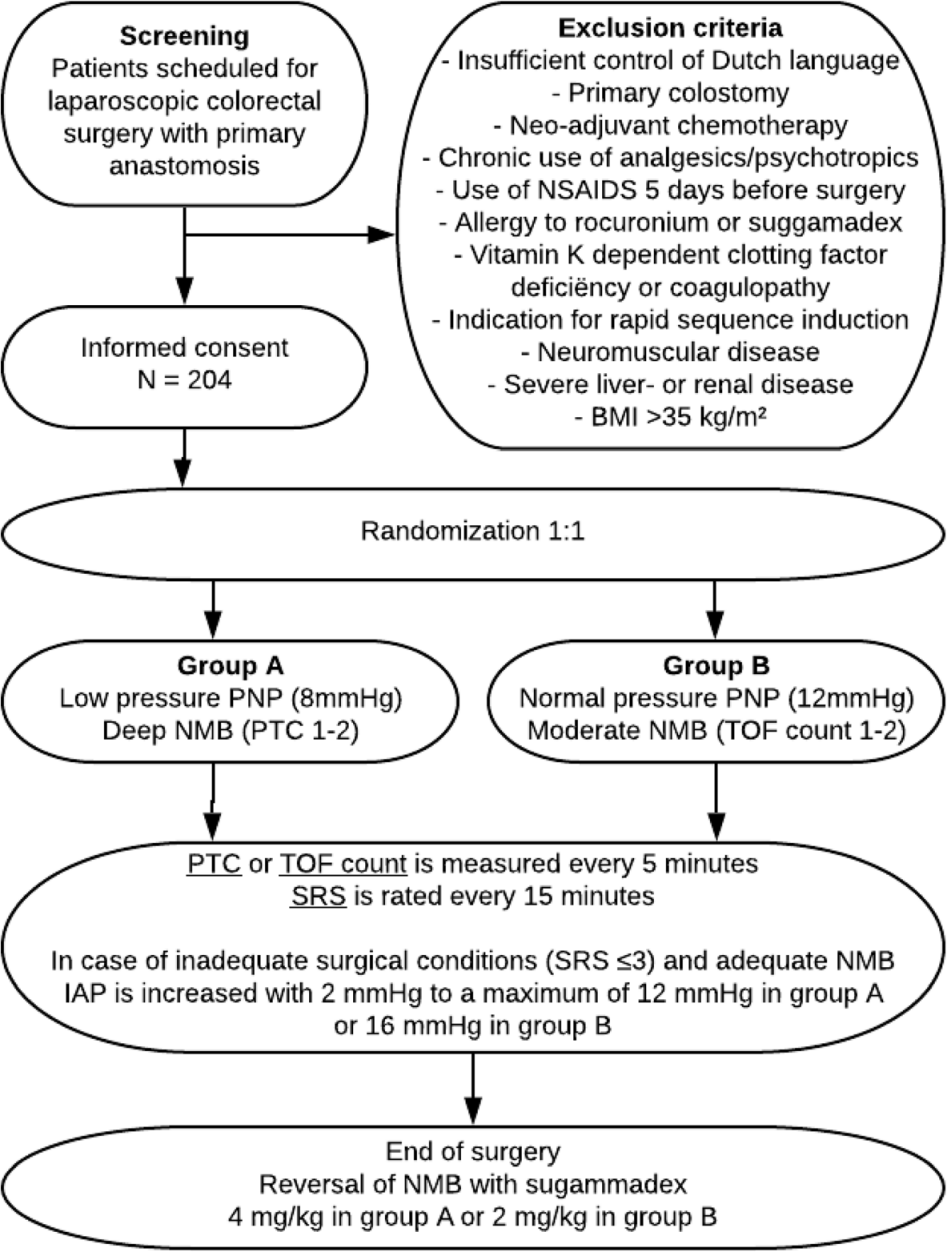
Study flow chart

The RECOVER study is a multicenter single-blinded randomized controlled trial that will be performed at three general teaching hospitals in the Netherlands. A list of participating centers is provided at the trial registration site (clinicaltrials.gov, NCT03608436). We aim to assess the effect of a lower pressure pneumoperitoneum facilitated by deep neuromuscular blockade on quality of recovery in patients undergoing laparoscopic colorectal surgery. All eligible patients will be screened and asked for informed consent. A flow chart of the inclusion process is shown in Fig. 1. Please see Additional files 1 and 2 for the SPIRIT figure and checklist, respectively.

### Study population

We aim to include 204 patients undergoing elective laparoscopic colorectal surgery. Adult individuals (≥ 18 years old) scheduled for laparoscopic colorectal surgery with a primary colonic anastomosis are eligible for this study. Patients will be excluded if they meet any of the following exclusion criteria: insufficient control of the Dutch language to understand patient information and fill out the questionnaires, primary colostomy, neo-adjuvant chemotherapy, chronic use of analgesics or psychotropic drugs, use of NSAIDs in the 5 days before surgery, known or suspected allergy to rocuronium or sugammadex, neuromuscular disease, indication for rapid sequence induction, severe liver or renal disease (creatinine clearance < 30 ml/min), morbid obesity (a body mass index > 35 kg/m^2^), or a deficiency of vitamin K-dependent clotting factors or coagulopathy, as sugammadex may increase the risk of bleeding in this group. Patients with a primary colostomy are not included in the study because this is likely associated with a significantly altered recovery process. Patients who have received neo-adjuvant chemotherapy are excluded because a substudy investigating immune function will be performed in a subgroup of the participants. Details of the substudy will not be further discussed here.

### Study protocol

After obtaining informed consent, patients will be randomized in a 1:1 fashion to group A, low-pressure pneumoperitoneum (8 mmHg) facilitated by deep neuromuscular blockade defined as a post tetanic count (PTC) of 1–2, or group B, normal-pressure pneumoperitoneum (12 mmHg) with moderate neuromuscular blockade defined as a train-of-four (TOF) count of 1–2. Randomization is supported by our statistician and will be performed by a randomization list created with Sealed Envelope [18]. Stratification by center and surgical technique (laparoscopic or robot assisted) will be used. Upon hospital admission and before surgery, patients will complete baseline questionnaires: the validated Dutch versions of the Quality of Recovery-40 [19] (QoR-40), McGill pain [20], and RAND-36 [21] questionnaires. In the participating hospitals, perioperative care is structured following the Enhanced Recovery After Surgery (ERAS) society guidelines for elective colorectal surgery [22, 23]. Adherence to the key elements of these guidelines will be scored for all patients.

In the operating room, the surgical team (surgeons and OR nurses) is blinded to the intervention allocation. The anesthesiologist and anesthesiologist assistant are not blinded in order to adequately monitor and regulate patient vitals and the level of neuromuscular blockade. A non-blinded physician researcher is present to oversee optimal execution of the study protocol. A standardized anesthesia protocol is used for all patients. General anesthesia will be induced with total intravenous anesthesia (TIVA) consisting of propofol 1–3 mg/kg and remifentanil 0.25–2 mcg/kg/min. After initiation of neuromuscular monitoring with the TofScan (an Equip Medikey [24] neuromuscular monitor using acceleromyography), an intubation dose of 0.6 mg/kg rocuronium (rocuroniumbromide, brand name Esmeron) is administered in both groups. After tracheal intubation, all patients are ventilated with pressure regulated volume controlled ventilation with a mixture of oxygen in air (ratio 1:3), 5 cm H_2_0 PEEP, and a tidal volume of 6–8 ml/kg. Respiratory rate is adjusted to reach an end-tidal carbon dioxide between 31 and 43 mmHg. Infusion of intravenous fluids is kept to a minimum, and losses will be replaced. If possible, the use of drains is avoided. When a nasogastric tube is indicated for gastric decompression, it will be removed before the end of surgery. Core temperature is continuously monitored and if necessary modified with a bair hugger temperature system, aiming at 36–37 °C.

General anesthesia is maintained with propofol aimed at a bispectral index score of 45–55, remifentanil 0.25–2 mcg/kg/min, a bolus injection of 0.2–1 mg/kg esketamine and a bolus injection of 1–1.5 mg/kg lidocaine 1% followed by continuous infusion of lidocaine 1% at 0.5–3 mg/kg/h. In both groups, the Automated TOF PTC (ATP) function of the TOF scan is used to measure the level of neuromuscular blockade every 5 min throughout the whole surgery. This function automatically determines whether a response to train-of-four stimulation is present and if TOF count or PTC should be measured. In group A (low pressure PNP with deep NMB), continuous infusion of 0.3–0.4 mg/kg rocuronium is initiated directly after intubation and titrated to a PTC of 1–2. If PTC is 0, continuous infusion will be decreased (but not stopped) until a PTC of 1 or 2 is reached. In group B (normal pressure with moderate NMB), a bolus or low dose of continuous rocuronium can be administered after intubation as normally done in clinical practice, titrating towards a TOF count of 1–2. Reaching deep NMB is carefully prevented in this group; if TOF count reaches 0, the infusion of rocuronium will be stopped until neuromuscular function is recovered.

After introduction of the camera trocar, the insufflation pressure of carbon dioxide is set to 8 mmHg in group A and 12 mmHg in group B, out of the surgical team’s sight. After introduction of the last trocar and every 15 min during surgery, a blinded OR nurse will ask the surgeon to rate the surgical conditions on the Leiden-Surgical Rating Scale (L-SRS) as displayed in Table 1. In case of inadequate surgical conditions (at any time during the surgery), defined as < 4 out of 5 points on the L-SRS scale, intra-abdominal pressure will be increased with 2 mmHg to 10 mmHg and a maximum of 12 mmHg in group A or 14 mmHg and a maximum of 16 mmHg in group B, respectively. If the level of NMB is not in the desired range, this will be corrected first. If surgical conditions remain compromised despite the increase in pressure, the surgeon can decide to convert to a hand-assisted or open procedure. All administered medication and intraoperative parameters are documented in the digital anesthesia report in the electronic patient file as in usual clinical practice. At the end of surgery, intraoperative complications will be registered by the surgeon.

After skin closure, NMB is reversed using sugammadex (natriumsugammadex, brand name Bridion), 4 mg/kg in group A and 2 mg/kg in group B, unless the TOF ratio in group B has spontaneously recovered to > 0.9. Extubation is performed when TOF ratio is stable at > 0.9 for 2 min and patients are fully awake. Postoperative pain management consists of acetaminophen and patient-controlled intravenous analgesia (PCIA) morphine or oxycodone in both groups.

### Outcome measures

The primary outcome of the study is the patient-reported outcome (PRO) *quality of recovery* on postoperative day 1 (24 h after surgery) measured with the validated Dutch version of the QoR-40 questionnaire. This survey consists of 40 short questions across five domains: physical comfort (e.g., nausea, dizziness or shivering), emotional state (general well-being, feeling anxious or angry), physical independence (ability to wash and groom), psychological support (from hospital staff, family and friends), and pain (presence of moderate pain, severe pain, and pain in several locations). Patients rate these aspects of recovery on a scale of 1 to 5, resulting in a total score between 40 and 200. Separate scores on each subdomain will also be explored. When the clinical condition of the patient allows it, they self-complete the questionnaires. If this is not the case, they will be assisted by a nurse blinded to the treatment allocation.

Intraoperative secondary outcome measures are quality of the surgical field quantified on the L-SRS every 15 min, estimated blood loss, pulmonary mean driving pressure, and intraoperative complications. Table 2 shows an overview of questionnaires and parameters collected at the post anesthesia care unit (PACU), surgical ward, and after discharge. Postoperative secondary outcomes are quality of recovery measured with the QoR-40 questionnaire after 72 h and 1 week; pain scores; postoperative nausea and vomiting (PONV); use of analgesics and anti-emetics after 1, 8, 24, and 72 h; intra-operative and postoperative complications up to 3 months after surgery (classified by the CLASS-Intra [25] and Clavien-Dindo [26] classifications, respectively); and length of hospital stay and time to reach discharge criteria. If patients are discharged before completion of the QoR-40 after 72 h or 1 week, the questionnaires are taken home and returned to the researchers by post. Pain is scored on a scale of 0–10 in rest and upon movement, if pain is acceptable yes or no and if shoulder pain is present yes or no. Nausea is scored on a scale of 0–10. Discharge criteria are as follows: adequate pain relief with oral analgesics, passage of flatus or defecation, intake of solid foods is tolerated, patient is capable of independent mobilization, and patient accepts discharge. The actual date of hospital discharge is also registered. Pain, nausea, and discharge criteria are scored by the ward nurse responsible for the clinical care of the patient, who is also blinded to the treatment allocation. Postoperative complications are extracted from the electronic patient file 1 month after surgery.

**Table 2:** Variables and time points

	− 1 day	1 h	8 h	24 h	48 h	72 h	1 week	1 month	3 months
**Questionnaires**									
QoR-40	X			X		X	X		
McGill pain	X								X
RAND-36	X								X
**Clinical parameters**									
Pain scores and PONV	X	X	X	X		X			
Analgesia and anti-emetics	X	X	X	X		X			
Complications				X	X	X	X	X	X
Discharge criteria			X	X	X	X			

### Adverse events and reactions

Adverse events are not expected, as the combined components of the intervention have demonstrated to be safe. As illustrated above, the use of deep neuromuscular blockade allows for a safe reduction in intra-abdominal pressure without compromising the surgical field. If surgical conditions are compromised nonetheless, the pressure will be increased to ensure patient safety at all times. The benefits of deep neuromuscular blockade are increasingly recognized in many types of surgery, with some reservation regarding reversal of different types of neuromuscular blocking agents and the risk of residual relaxation [27]. Sugammadex is able to safely reverse prolonged rocuronium-induced deep neuromuscular block, with no recurrence of blockade [28, 29]. To prevent an increased risk of pulmonary complications, neuromuscular monitoring will strictly be applied as specified in the ERAS Society Recommendations for perioperative care in elective colorectal surgery [30]. A recent retrospective study by Boon and colleagues even shows that compared to low dose, high-dose rocuronium is associated with a lower incidence of unplanned 30-day readmissions than low-dose rocuronium [31]. Patients will be observed at the PACU to confirm smooth recovery from anesthesia.

### Statistical methods and sample size calculation

The mean clinically important difference on the QoR-40 questionnaire as described by Myles et al. is 6.3 points on a scale of 40–200, with a standard deviation of 15 points [32]. In order to achieve 80% power to detect a 6.3 point difference with an *α* of 5%, a sample size of 89 patients per group is needed. Considering a 15% conversion rate to open surgery, 204 patients are required to secure 178 patients for the final analysis.

For the primary outcome analysis, factorial ANCOVA will be used to compare the QoR-40 score on postoperative day 1 for groups A and B and adjusted for covariates. For secondary outcome variables, a Student’s *t* test will be used to compare normally distributed variables, and a Mann-Whitney *U* test will be used for skewed variables. For categorical variables, a chi-square test will be performed. A *P* value of < 0.05 will be considered statistically significant. All analyses will be performed on an intention to treat base. For exploratory outcome measures, Bonferroni correction for capitalization on chance will be applied.

### Data management and monitoring

All patient data will be coded and stored anonymously in the certified cloud-based electronic data collection platform Castor [33]. A subject identification list is kept separate and securely stored in compliance with privacy legislation. Monitoring will be performed according to the negligible risk guidelines of the Dutch federation of academic medical centers [34]. A yearly progress and safety report will be submitted to the medical research ethics committee and competent authority.

## Discussion

One of the most rewarding collaborations between surgeons and anesthesiologists resulted in the development of the international evidence-based Enhanced Recovery After Surgery (ERAS) guidelines, introducing a cost-effective decrease in length of hospital stay and complications after surgery. The advantages vary between different types of surgery; however, the ERAS guideline for colorectal surgery is currently well established as the optimal standard of care [35]. Further optimization of surgical conditions requires a continued partnership in the operating room, where a promising advancement lies in the relationship between intra-abdominal pressure and the degree of muscle relaxation. There is extensive evidence to support that the pressure used to create a pneumoperitoneum during laparoscopic surgery is harmful to surrounding organs and structures. Insufflation pressures lead to compression of the capillary vasculature, causing ischemia-reperfusion injury and oxidative stress, especially during prolonged exposure [36–38]. Perfusion of the parietal peritoneum is significantly improved at low-pressure compared to standard pressure pneumoperitoneum (*Albers* et al.*, submitted*). Deep neuromuscular block reduces the pressure requirements and increases intra-abdominal volume, thereby increasing the available surgical workspace at a lower intra-abdominal pressure [39, 40]. Reducing intra-abdominal pressure without deep muscle relaxation compromises the surgical workspace and may not be safe, as illustrated by a higher incidence of surgical complications in patients with moderate neuromuscular block and low pressure pneumoperitoneum during our previous study in patients undergoing laparoscopic donor nephrectomy [9]. In addition, deep neuromuscular blockade in itself appears to be beneficial, leading to reduced postoperative pain scores and a decrease in opiate consumption independent of insufflation pressures [9, 41]. We confirmed these findings in our recently completed trial comparing moderate to deep neuromuscular block during laparoscopic surgery in living kidney donors [42]. Therefore, we hypothesize that the combined benefits of lower intra-abdominal pressure and deep neuromuscular blockade can further enhance quality of recovery after laparoscopic surgery.

Even though the beneficial effects of lower intra-abdominal pressure and deep neuromuscular blockade on surgical conditions are increasingly recognized for many laparoscopic procedures, the results on patient outcomes—especially for colorectal procedures—largely remain to be elucidated. The QoR-40 questionnaire provides a validated, reliable assessment of early quality of recovery [17]. The participating centers for this trial have been selected on satisfactory to exemplary compliance with the ERAS guidelines, Martini general hospital is one of the 24 ERAS centers of excellence worldwide [43]. This will allow us to determine whether low pressure pneumoperitoneum facilitated by deep neuromuscular blockade could be a valuable addition to the ERAS program. One of the main strengths of our study is that close registration of the perioperative parameters will allow for a reliable examination of the true effects of low pressure and deep neuromuscular blockade during colorectal laparoscopic surgery. A limitation of the study is that it will be performed in a single blinded manner; only the surgical team will be blinded. Blinding the anesthesiologist is not feasible as this would impair patient safety. After extensively weighing the costs and benefits, we chose not to blind the researcher in the operating room to improve adherence to the study protocol, especially regarding neuromuscular blockade. In our previous trials in living kidney donors, we have observed that achieving and maintaining adequate deep neuromuscular blockade throughout the whole surgery remains challenging. This resulted in cases with recurrent recovery to moderate or even shallow NMB during the procedure, disrupting the intended analysis. Currently, there are no clear guidelines concerning adequate dose and monitoring for a well maintained deep NMB. Moreover, there is a large variability in reported doses between trials aiming for a similar or equal block depth, and corresponding monitoring data is often lacking. By allowing the focus of one competent physician researcher on dosing and monitoring, we strive for less fluctuations and a representative intervention across all patients. To diminish the risk of bias, (post) operative outcome measures such as L-SRS scores, complications, and questionnaires will be registered and collected by a blinded member of the surgical team and blinded research nurse, respectively. Additionally, we strongly advocate the importance of developing a consensus guideline for deep neuromuscular blockade in future trials and use in clinical practice.

In conclusion, this multicenter randomized clinical trial will investigate the effect of low pressure PNP facilitated by deep NMB on quality of recovery after laparoscopic colorectal surgery. Moreover, the study will identify whether low pressure PNP and deep NMB could be a valuable addition to the ERAS guideline for colorectal surgery.

### Trial status

Protocol version 2—May 2018. Recruitment began in October 2018, and predicted completion of inclusion is in October 2020.

## Supplementary information

**Additional file 1.** SPIRIT figure. Schematic overview of enrolment, interventions, and assessments.

**Additional file 2.** SPIRIT 2013 Checklist: Recommended items to address in a clinical trial protocol and related documents.

## Data Availability

All relevant data will be included in the article and its supplementary information files. Additional information is available from the corresponding author on reasonable request.

## Abbreviations

ANCOVA: Analysis of covariance
ERAS: Enhanced Recovery After Surgery
L-SRS: Leiden-Surgical Rating Scale
NMB: Neuromuscular blockade
NMBA: Neuromuscular blocking agent
OR: Operating room
PNP: Pneumoperitoneum
POD1: Postoperative day 1
PTC: Post tetanic count
TOF: Train of four
QoR-40: Quality of Recovery-40
SPIRIT: Standard Protocol Items: Recommendations for Interventional Trials
