# Supplementary material for: The effect of low- versus normal-pressure pneumoperitoneum during laparoscopic colorectal surgery on the early quality of recovery with perioperative care according to the enhanced recovery principles (RECOVER): study protocol for a randomized controlled study
Source: Trials. 2020 Jun 17;21:541. doi: 10.1186/s13063-020-04496-8 (PMC7301516; doi:10.1186/s13063-020-04496-8)
Supplement: Supplementary file 1 — Additional file 1. SPIRIT figure. Schematic overview of enrolment, interventions, and assessments. [file 13063_2020_4496_MOESM1_ESM.doc]

SPIRIT figure. Schematic overview of enrolment, interventions, and assessments

|  | | **STUDY PERIOD** | | | | | | | | | | |
| --- | --- | --- | --- | --- | --- | --- | --- | --- | --- | --- | --- | --- |
|  | | **Enrolment** | | **Allocation** | **Post-allocation** | | | | | | | **Close-out** |
| **TIMEPOINT** | | ***-2 weeks*** | ***-1 day*** | ***Day of surgery*** | ***1h*** | ***8h*** | ***24h*** | ***48h*** | ***72h*** | ***1 week*** | ***1 month*** | ***3 months*** |
| **ENROLMENT:** | |  |  |  |  |  |  |  |  |  |  |  |
| **Eligibility screen** | | X |  |  |  |  |  |  |  |  |  |  |
| **Informed consent** | | X |  |  |  |  |  |  |  |  |  |  |
| **Allocation** | |  |  | X |  |  |  |  |  |  |  |  |
| **INTERVENTIONS:** | |  |  | During surgery |  |  |  |  |  |  |  |  |
| ***A*** | ***IAP 12 mmHg moderate NMB*** |  |  |  |  |  |  |  |  |  |  |  |
| ***B*** | ***IAP 8 mmHg***  ***Deep NMB*** |  |  |  |  |  |  |  |  |  |  |  |
| **ASSESSMENTS:** | |  |  |  |  |  |  |  |  |  |  |  |
| ***Baselines characteristics*** | |  | X |  |  |  |  |  |  |  |  |  |
| ***L-SRS*** | |  |  |  |  |  |  |  |  |  |  |  |
| ***Questionnaires*** *- QoR-40 - McGill pain - RAND-36* | |  |  |  |  |  |  |  |  |  |  |  |
|  |  |  | X |  |  |  | X |  | X | X |  |  |
|  |  |  | X |  |  |  |  |  |  |  |  | X |
|  |  |  | X |  |  |  |  |  |  |  |  | X |
| ***Pain scores*** | |  | X |  | X | X | X |  | X |  |  |  |
| ***Nausea, vomiting*** | |  | X |  | X | X | X |  | X |  |  |  |
| ***Analgesia use*** | |  | X |  | X | X | X |  | X |  |  |  |
| ***Discharge criteria*** | |  |  |  |  | X | X | X | X |  |  |  |
| ***Complications*** | |  |  |  |  |  | X | X | X | X | X | X |

IAP = intra-abdominal pressure; NMB = neuromuscular blockade; L-SRS = Leiden-Surgical Rating Scale; QOR-40 = Quality of Recovery-40 questionnaire; RAND-36 = Research ANd Development-36 questionnaire.
